# Changes in women’s physical function in mid-life by reproductive age and hormones: a longitudinal study

**DOI:** 10.1186/s12905-022-02070-9

**Published:** 2022-11-24

**Authors:** Fanny Kilpi, Ana Goncalves Soares, Gemma L. Clayton, Abigail Fraser, Paul Welsh, Naveed Sattar, Scott M. Nelson, Kate Tilling, Deborah A. Lawlor

**Affiliations:** 1 MRC Integrative Epidemiology Unit, University of Bristol, Oakfield House, Oakfield Grove, Bristol, BS8 2BN UK; 2 Population Health Sciences, Bristol Medical School, Bristol, UK; 3 Bristol NIHR Biomedical Research Centre, University Hospitals Bristol NHS Foundation Trust and University of Bristol, Bristol, UK; 4 Institute of Cardiovascular and Medical Sciences, University of Glasgow, Glasgow, UK; 5 School of Medicine, Dentistry and Nursing, University of Glasgow, Glasgow, UK

**Keywords:** ALSPAC, Physical function, Menopause, Reproductive age, Reproductive hormones

## Abstract

**Background:**

Whether women’s physical function in mid-life is related to their reproductive age is not known. The objectives of this study were to examine and compare changes in physical function in women by reproductive age, measured as time since final menstrual period (FMP), and chronological age, and to explore associations with repeatedly assessed levels of reproductive hormones.

**Methods:**

We used data from 2319 UK women with up to three repeated measurements of physical function (median length of follow up: 2 years), focusing on changes occurring in women experiencing a natural menopausal transition. The main outcome was a composite physical function score that incorporated assessments of strength (grip strength), balance (one-leg stand) and cardiorespiratory fitness (timed chair rises). Associations with time since FMP, age, and time-updated measures of anti-Müllerian hormone, follicle-stimulating hormone and luteinizing hormone were assessed by multilevel models and generalised estimating equations models adjusted for the underlying effects of chronological age and confounding by education, age at first birth and smoking.

**Results:**

The results showed that, adjusted for these confounders, time since FMP (− 0.21 SD per 10 years, 95% CI − 0.37, − 0.06) and chronological age (− 0.31 SD per 10 years, 95% CI − 0.46, − 0.15) were inversely associated with the physical function composite score. Grip strength seemed to be the main contributor to the decline in the composite score by time since FMP. There was no strong evidence of associations between any of the three reproductive hormones and the composite score.

**Conclusions:**

Physical function in women in mid-life declined with both chronological and reproductive age. The decline with reproductive age was independent of chronological age but did not seem to be driven by changes in reproductive hormones.

**Supplementary Information:**

The online version contains supplementary material available at 10.1186/s12905-022-02070-9.

## Background

Maintaining a high level of physical function is key for accomplishing daily activities and supporting a sense of independence. Physical function measures such as grip strength and walking speed are also inversely associated with disability and mortality risk [1–4]. Physical function declines from mid-life [5, 6], and performance on tests of physical function is lower on average in women than men of the same age [6]. Some studies indicate sex differences in the rate of decline in physical function [6–9], which could result from differences in body composition, comorbidities, and health-related behaviours. Changes occurring across the menopausal transition in women, such as hormonal influences on body composition, may also play a role for women’s physical function [10–14], but this is not yet well understood.

Most evidence on women’s physical function in the menopausal transition has been based on cross-sectional analyses [10, 15–20], and has indicated that women have a lower grip strength in peri- and post-menopausal stages than in pre-menopause [10, 18, 20]. However, these findings may reflect general ageing effects, as cross-sectional studies are less able to differentiate between reproductive ageing, chronological ageing, and potential cohort effects. Only a few longitudinal studies have used objective measures of physical function [21, 22]. One study with a five-year follow-up measured walking speed, strength, and flexibility up to four times in 530 women in the US [22] and found that, adjusted for time, body mass index (BMI) and smoking, post-menopausal women had lower performance in walking speed and lifting, and experienced a greater decline in grip strength compared with pre-menopausal women. Another US study with a three-year follow-up (N = 485) adjusted for age and measured confounders, and found that transitioning from pre-menopause to late peri- or post-menopause was negatively associated with grip and pinch strength [21].

As the existing longitudinal studies have examined menopausal stage, but not the hormonal changes that accompany reproductive ageing, the aim of this study was to examine and compare the associations of reproductive age measured by time since final menstrual period (FMP), chronological age and repeatedly assessed levels of anti-Müllerian hormone (AMH), follicle-stimulating hormone (FSH), and luteinizing hormone (LH) with physical function in mid-life. We used repeat assessments of objective physical function in a large longitudinal study from a general population sample and explored the relationship of physical function with reproductive hormones that reflect menopause transition related changes.

## Methods

The study is based on the follow up of women originally recruited when pregnant with an expected delivery date between 1st April 1991 and 31st December 1992 in the area of Bristol in the South West of England [Avon Longitudinal Study of Parents and Children (ALSPAC)]. The initial number of pregnancies enrolled was 14,541 and the families have been followed up with regular assessments to the present day [23, 24]. The study website (http://www.bristol.ac.uk/alspac/researchers/our-data/) contains details of all the data that is available through a fully searchable data dictionary and variable search tool.

**Fig. 1:**
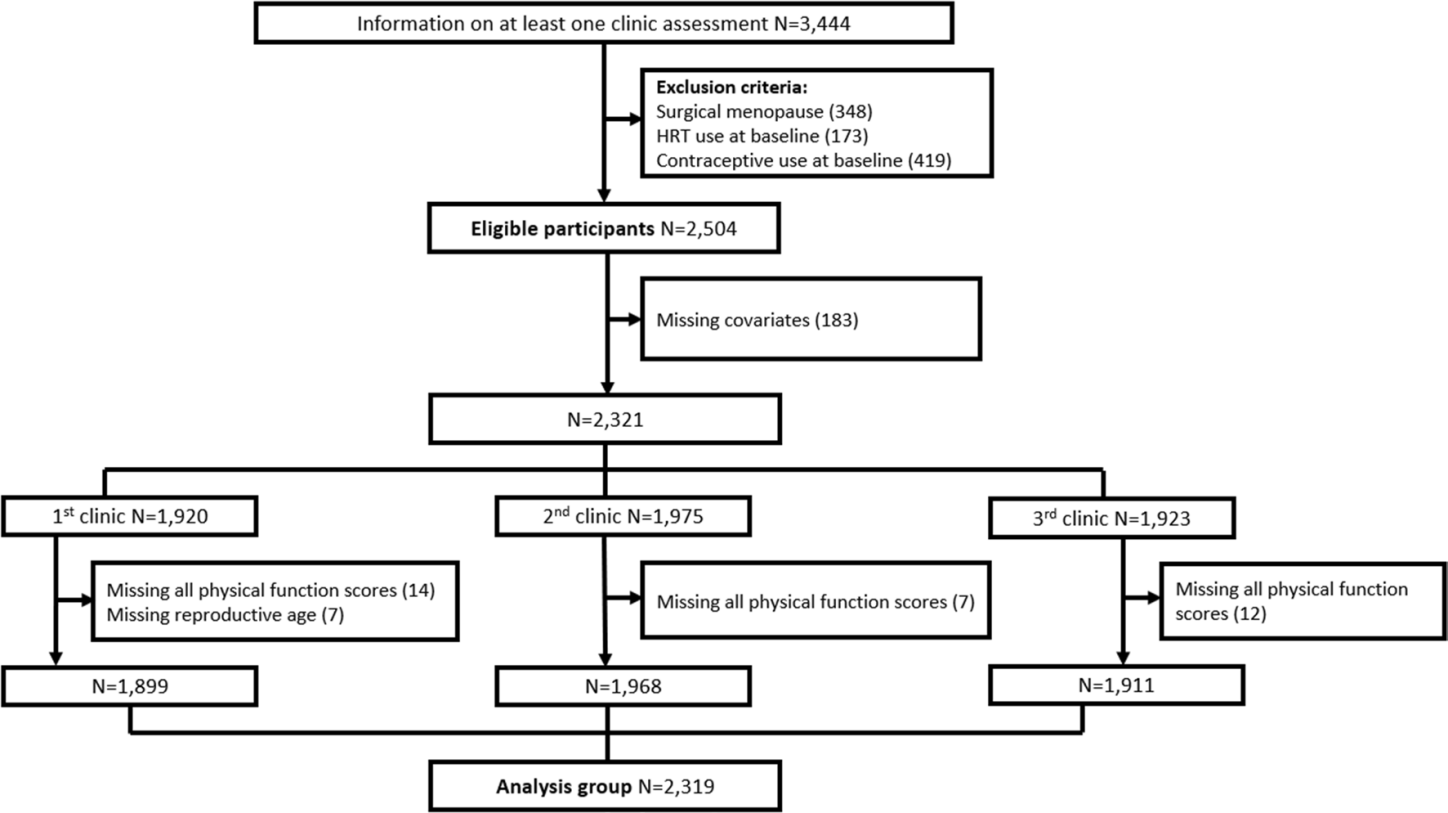
Participant flow

Mothers who were actively participating in the study in their mid-life were invited to take part in repeated clinic assessments, which had the aim of studying the health and lifestyle changes occurring during mid-life and the menopausal transition [23, 25, 26]. Our analysis sample is based on women who participated in at least one of three clinic assessments taking place between 2011 and 2015, when tests of physical function were administered. Women who had undergone surgical menopause (i.e. hysterectomy, oophorectomy, endometrial ablation, or radio- or chemotherapy related to reproductive organs) at baseline or follow-up were excluded, as were women reporting using hormone replacement therapy (HRT) or hormonal contraception at baseline, so that the focus was on changes occurring across a natural menopause transition. Observations for women who reported using HRT or hormonal contraception in the follow-up were censored at the last point before reported use. The participant flow for the main analyses (N = 2319) is described in Fig. 1. The number of participants contributing one, two or three follow up visits was 512, 454 and 1353, respectively.

### Exposures

Participants were asked a detailed set of questions about their menstrual periods at each clinic assessment, including the date of their last period and their regularity. We established the date of the FMP based on the self-reported date of the last period after which the participant had experienced amenorrhea for at least 12 months. Time since FMP (in years), was calculated retrospectively and coded zero for pre-FMP observations, because there was a relatively low number of participants who experienced their FMP during the follow-up to allow for meaningful analyses of change in years up to the FMP. We kept these participants in the analyses, as excluding them would limit the analysis to older women and weight the analysis to later reproductive ages. Chronological age was calculated from date of clinic attendance and self-reported date of birth and centered at 50.

Concentrations of reproductive hormones AMH, FSH and LH were assayed in serum from venous blood samples taken at each clinic. The samples were immediately centrifuged and frozen at − 80 °C until thawed for hormonal analyses (with no previous thaw-freeze cycles). AMH was measured using the fully automated Elecsys AMH Plus immunoassay [27], and FSH and LH with a Roche Elecsys modular analytics Cobas e411 using an electrochemiluminescence immunoassay. The hormones were treated as time-varying variables and were standardized using the mean and standard deviation (SD) from the first clinic assessment.

### Confounders

Based on their potential effect on age at menopause and physical function we considered socioeconomic position (SEP), age at first birth, smoking and BMI to be potential confounders. Education was used as a measure of SEP and was measured at recruitment (to the original cohort) by the self-reported highest attained qualification: (1) Certificate of Secondary Education, ordinary O-level or vocational certificate (qualifications usually obtained at age 16, the UK minimum school leaving age when these women were at school), (2) Advanced A-level (usually taken at 18 years) or (3) university degree. Self-reported age at first birth was also obtained at the time of the recruitment to the original study and was centered at 26 years. Smoking status was derived from questionnaires before the first of the women’s mid-life repeat clinic assessments, and coded as (1) never smoker, (2) former smoker or (3) current smoker. BMI (kg/m^2^) was measured at all clinic assessments. Weight and height were measured in light clothing and without shoes. Weight was measured to the nearest 0.1 kg using Tanita scales and height to the nearest 0.1 cm using a Harpenden stadiometer.

### Outcomes

Our primary outcome is a composite score of physical function which has been developed to provide an overall measure of physical function [28]. The measure incorporates an assessment of strength, balance and cardiorespiratory fitness from three tests that we administered at each clinic: (1) a test of maximum grip strength (kg) using a Jamar handgrip dynamometer (strength); (2) timed one-leg stand with eyes closed (in seconds, maximum 30 s; balance); (3) a test of how long it takes to complete ten chair rises from sitting to standing (in seconds; strength, balance and cardiorespiratory fitness). Scores on each of these tests were rescaled to lie between zero (lowest performance) and one (best performance) and then summed to give the composite score (details can be found in Additional file 1: Supplementary Text). We standardised the composite score by subtracting the mean from the first clinic and dividing by the estimated between-individual SD from the adjusted model described below. We completed two additional tests: the timed one-leg stand with eyes open and a timed three-meter walk, as further tests of balance and cardiorespiratory fitness.

As secondary outcomes, we examined each test individually. Maximum grip strength (kg) was treated as a continuous variable. Due to their non-normal distributions, we transformed scores on the other tests into binary variables characterising low performance: standing less than 30 s in the one-leg stand with eyes open (based on most participants achieving the maximum), less than 3 s in the one-leg stand with eyes closed (the limit of lowest quartile in the first clinic), walking speed slower than 1.1 m/s (lower 95% confidence interval limit of walking speed for women aged 50–59 [29]) and taking more than 26 s to complete 10 chair rises (based on lowest quartile in the first clinic).

### Analysis strategy

We used multilevel linear regression models (MLM) to model the composite physical function score and grip strength. Generalised estimating equations (GEE) with a logit link, unstructured correlation matrix and robust standard errors were used to model the binary outcomes. We examined associations with physical function by relating the outcomes to: (1) time since FMP (with a random intercept and a random slope in MLM models) and a dummy variable for whether the observation was pre-FMP; (2) chronological age (with a random intercept and a random slope); (3) mutually adjusted chronological age and time since FMP (with a random intercept and a random slope for both in MLM models); and lastly, (4) additionally adjusted for education, age at first birth and smoking. The associations with hormone exposures were examined in two models: (1) unadjusted (with a random intercept in MLM models); and (2) adjusted for chronological age (with a random intercept and a random slope in MLM models), education, age at first birth and smoking.

### Sensitivity analyses

In sensitivity analyses, we additionally adjusted the main models for baseline and time-varying BMI, because BMI may be both a confounder and a mediator of the association between reproductive age and physical function. In addition, because preliminary analyses indicated small improvements in performance for some measures in the follow-up, potentially reflecting practice effects (e.g. increasing familiarity and ease in performing the administered tests), we performed analyses additionally controlling for a dummy variable for prior test exposure. The main analyses were also repeated restricting the sample to women who participated in all three clinics (N = 1353). To make full use of available follow-up data, the main analyses were repeated in data that additionally included repeat measurements from the re-invitations to the clinic of small random subsamples of participants after each clinic that had the original purpose of examining measurement reliability (participants were required to live locally and have gone through their original visit in standard order to be re-invited). In our sample, 161 women had these additional repeat measurements on average 2 months after the main clinic. Finally, we examined how physical function was associated with menopausal stage across chronological age in GEE models adjusted for menopausal stage, chronological age, and their interaction, and education, age at first birth and smoking. Pre-, peri- and post-menopause were categorised according to Stages of Reproductive Aging Workshop (STRAW) criteria [30].

## Results

The mean age of the participating women was 50.5 in the first clinic assessment and 52.8 by the last (Table 1) with a median duration of follow-up of 2.1 years. 38.6% of women at the first clinic assessment were premenopausal, which decreased to 21.7% by the end of follow-up. Average performance on grip strength, walking and chair rise tasks improved slightly in the follow-up, likely due to practice effects, and declined for the one-leg stand tests.

**Table 1:** Characteristics of participants by clinic assessment

	1st clinic (N = 1899)	2nd clinic (N = 1968)	3rd clinic (N = 1911)
Age, mean (SD)	50.5 (4.4)	51.7 (4.4)	52.8 (4.3)
Years since FMP, mean (SD)	1.9 (3.4)	2.5 (3.8)	3.2 (4.2)
AMH (ng/ml), median (IQR)	0.01 (0.01–0.17)	0.01 (0.01–0.08)	0.01 (0.01–0.03)
FSH (mIU/ml), median (IQR)	36.38 (7.56–74.88)	57.97 (11.80–88.08)	64.16 (19.04–89.14)
LH (mIU/ml), median (IQR)	24.55 (7.40–40.11)	31.49 (10.56–44.58)	31.86 (15.96–43.28)
*Menopausal stage*			
Pre-menopause, % (N)	38.6 (720)	29.0 (523)	21.7 (376)
Peri-menopause, % (N)	27.2 (507)	28.3 (511)	26.8 (465)
Post-menopause, % (N)	34.1 (636)	42.7 (769)	51.4 (891)
*Physical function*			
Physical function composite score, mean (SD)	1.34 (0.37)	1.40 (0.37)	1.39 ( 0.37)
Grip strength (kg), mean (SD)	26.04 (6.61)	27.68 (6.09)	28.03 ( 6.44)
Inability to complete 30 s one-leg stand eyes open, % (N)	15.9 (299)	12.8 (249)	18.9 (354)
Inability to complete 3 s one-leg stand eyes closed, % (N)	25.2 (473)	31.3 (608)	34.0 (636)
Walking speed less than 1.1 m/s, % (N)	25.2 (474)	19.8 (388)	17.2 (325)
Inability to complete 10 chair rises in 26 s, % (N)	26.0 (478)	17.2 (329)	15.3 (283)
*Education*			
CSE/vocational/O-level, % (N)	45.9 (871)		
A-level, % (N)	30.4 (578)		
Degree, % (N)	23.7 (450)		
*Smoking status*			
Never smoker, % (N)	56.3 (1069)		
Former smoker, % (N)	35.9 (682)		
Current smoker, % (N)	7.8 (148)		
Age at first pregnancy, mean (SD)	26.7 (4.7)		
BMI, mean (SD)	26.2 (5.0)	26.3 (5.2)	26.2 (5.3)

*AMH* anti-Müllerian hormone, *BMI* body mass index, *CSE* certificate of secondary education, *FMP* final menstrual period, *FSH* follicle-stimulating hormone, *LH* luteinizing hormone, *O-level* ordinary level, *OR* odds ratio, *SD* standard deviation

**Fig. 2:**
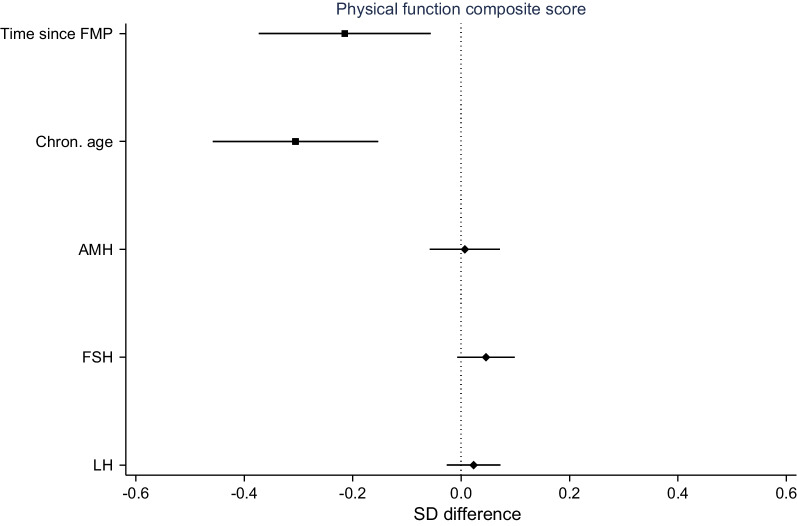
Adjusted estimates of composite physical function by time since FMP, chronological age, and reproductive hormones. *Note*: Effect estimates for time since FMP (by 10 years), chronological age centered at 50 (by 10 years), and reproductive hormones from models adjusted for age, education, age at first birth and smoking. Positive effect estimates reflect better functioning. *AMH* anti-Müllerian hormone, *FMP* final menstrual period, *FSH* follicle-stimulating hormone, *LH* luteinizing hormone, *SD* standard deviation

Time since FMP and chronological age were both inversely associated with the composite score in the adjusted model (Fig. 2, Additional file 1: Table S1). In unadjusted models, the fit of time since FMP and chronological age were similar in terms of Bayesian Information Criterion (BIC) (Additional file 1: Table S2). There was no strong evidence of associations between AMH, FSH and LH and the composite score, with point estimates being close to the null (Fig. 2, Additional file 1: Table S3).

**Fig. 3:**
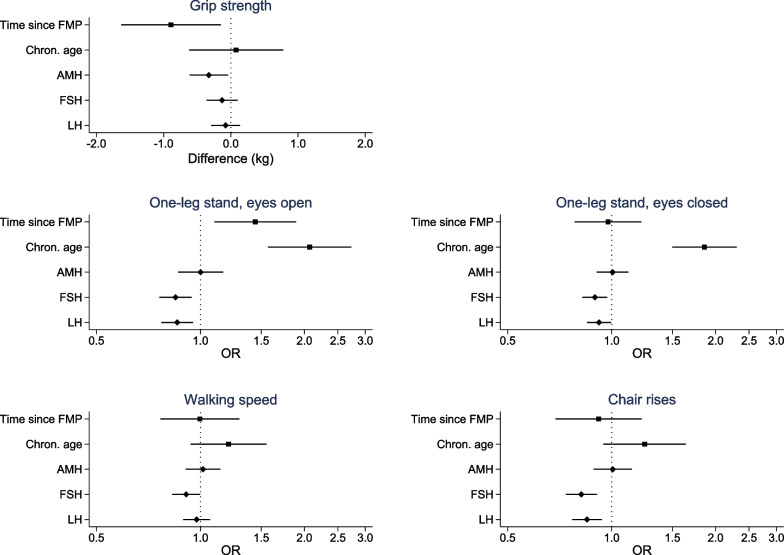
Adjusted estimates of physical function outcomes by time since FMP, chronological age, and reproductive hormones. *Note*: Effect estimates for time since FMP (by 10 years), chronological age centered at 50 (by 10 years), and reproductive hormones from models adjusted for age, education, age at first birth and smoking. Positive effect estimates for grip strength reflect better functioning, whereas positive OR for one-leg stand, walking speed and chair rises indicate worse functioning. *AMH* anti-Müllerian hormone, *FMP* final menstrual period, *FSH* follicle-stimulating hormone, *LH* luteinizing hormone, *OR* odds ratio

Results for the individual tests were inconsistent. In adjusted models, time since FMP was associated with lower grip strength and poorer performance on the one-leg stand eyes open test (Fig. 3, Additional file 1: Table S2), but not with the other tests. Therefore, grip strength was the main contributor to the decline by time since FMP in the composite score. Chronological age was associated with poor performance on both one-leg stand tests, and weakly associated with poor performance on the walking speed and chair rise tests, but not with grip strength.

Associations of reproductive hormones with physical performance measures were also inconsistent. A higher level of AMH, indicative of pre-menopause, was associated with weaker grip strength (Fig. 3, Additional file 1: Table S3), but no other physical function test. Higher levels of FSH and LH, indicative of the menopausal transition, were associated with better performance on both the one-leg stand tests, chair rises and, and FSH was also associated with walking speed.

### Results of sensitivity analyses

Analyses adjusting for baseline or time-varying BMI (Additional file 1: Figs. S1, S2, Tables S1, S2, S3) yielded similar results to the main adjusted models. Adjusting for previous test exposure slightly attenuated the point estimates of the associations of time since FMP with physical function composite score and grip strength and increased the inverse association of chronological age with nearly all physical function measures (Additional file 1: Fig. S3).

Women who participated in all three clinic assessments were not meaningfully different at baseline to those lost to follow-up (Additional file 1: Tables S4, S5) and results of analyses limited to women who participated in all clinic assessments were very similar to the main analyses but with wider confidence intervals (Additional file 1: Fig. S4, Tables S6, S7). Results were also similar in the data incorporating clinic re-invitations data (Additional file 1: Figs. S5, S6, Table S8).

When examining the association between chronological age and physical function across menopausal stages (Additional file 1: Fig. S7, Table S9), the decline in physical function by age was somewhat steeper in post-menopause (− 0.48 SD, 95% CI − 0.68, − 0.28) compared with pre-menopause (− 0.31 SD, 95% CI − 0.55, − 0.06), which agreed with the additional impact of time since FMP in the main analyses. Grip strength was lower in post-menopause compared with pre-menopause (− 0.85 kg 95% CI − 1.47, − 0.23), but menopausal stage had a limited impact on the decline by age. For the one-leg stands and chair rises, performance tended to be similar, or slightly better, across successive menopausal stages, whereas decline by age was greater in post-menopause. For walking speed, the predicted probability of low performance increased more steeply with age in peri-menopause than pre-menopause (OR 2.25, 95% CI 1.34, 3.76, vs. OR 1.05, 95% CI 0.68, 1.63).

## Discussion

Physical function as indicated by a combined measure of objectively assessed muscle strength, balance, and cardiorespiratory fitness, declined in women in mid-life in relation to both chronological age and reproductive age. However, the analyses of three reproductive hormones did not indicate that these explained any of the decline with reproductive age.

Our results partly support previous evidence [10, 18, 20–22], largely from cross-sectional studies and studies that have primarily focused solely on muscle strength, of an inverse association between reproductive age and physical function. Our study adds considerably to these previous studies by exploring a primary composite outcome which considers three key aspects of physical function and by modelling repeatedly assessed function across reproductive age and in relation to reproductive hormones. Our results for chronological age correspond to findings of decreasing overall physical function, grip strength, and performance on chair rises, gait speed and leg stand tests in women with age [6, 9, 31–34]. That we confirm previous studies of declining physical function with age suggests our study sample may generalise to other populations. However, sensitivity analyses adjusting for previous test exposure suggested that such decline may have been underestimated in our main analysis. The closely spaced follow-up visits may have resulted in some improvement by practice or test familiarity, but with a relatively small number of repeat measures there is some uncertainty.

The results for our primary outcome indicated that greater time since FMP was also associated with lower overall physical function when adjusted for chronological age and confounders. This decline may reflect hormonally-driven changes in BMI and muscle, bone, and fat mass in post-menopausal women [11–14, 35]. Adjusting for baseline or time-varying BMI had little effect on the point estimates, and therefore was unlikely to be a major confounder or mediator. Few previous studies have had repeated performance-based measures, and those that have, focused on menopausal stages, and had substantially smaller, younger samples with fewer postmenopausal women than in our study [21, 22]. Similar to our main results, they found decrements in grip strength in post-menopausal women. When we examined the decline with age at different menopause stages, the composite score decreased more steeply in post-menopause than pre-menopause, but this was not evident for grip strength. Nevertheless, grip strength was lower in post-menopause than in pre-menopause.

By examining both reproductive age and hormones, we aimed to explore whether any changes with reproductive age reflect hormonal changes that occur as women transition through the menopause. We were unable to study the relationship with oestrogen, but had repeated measures of FSH and LH, which show a pattern of increase from 2 years prior to 2 years after FMP [30, 36, 37], mirroring the decrease in oestrogen. We found that the associations of hormones with the individual tests did not always follow the direction anticipated by the association with reproductive age. The most marked changes in FSH, LH and AMH occur just before or during the menopausal transition [36–38], whereas time since FMP reflects post-menopausal reproductive ageing. This may explain the seemingly inconsistent findings for reproductive age and hormone levels. Furthermore, evidence from a recent meta-analysis of twelve studies indicated that HRT in postmenopausal women had a limited effect on muscle mass [39], but an earlier meta-analysis of HRT and muscle strength suggested a small positive association [40].

### Strengths and limitations

To our knowledge, this is the largest longitudinal study published to date using objective measures of physical function to examine how reproductive age and hormones are associated with physical function in women with a natural menopausal transition. The use of a composite score of the key physical function elements of strength, balance, and cardiorespiratory fitness, and exploring associations with each of these is also a strength over previous studies that have largely explored only grip strength. The longitudinal study design means that the effect estimates for continuous outcomes better reflect within-individual changes in comparison to a cross-sectional study. As we simultaneously adjust for chronological age, the effect estimates for reproductive age and hormones are best interpreted as mean differences between two women of the same age with a unit difference in reproductive age/hormones. The measure of reproductive age, time since FMP, was coded as zero before the menopause due to the relatively low number of women observed to experience their FMP during the study follow-up (N = 165), as calculating reproductive age with negative values prior to the FMP would have resulted in women who remained pre-FMP (N = 1128) being excluded from the analyses. Our median and maximum follow ups (2.1 and 3.5 years, respectively) were shorter than the menopausal transition takes on average, [30] and having data across the menopausal transition for the complete sample would be advantageous. With a maximum of three repeated measures of physical function, we were also limited in our ability to explore whether reproductive age or hormones were associated with non-linear patterns of change in physical function. Despite being one of largest studies to explore these associations, further studies with a larger proportion of women experiencing menopause during a longer follow-up would be valuable to analyse in detail the potential changes in physical function in the years just before and after the FMP.

We aimed to focus on changes in physical function in women experiencing a natural menopause. However, studies have found older age at menopause to be associated with better physical function [10, 41], and thus if age at menopause or its determinants are causally associated with physical function, it may act as a confounder. We therefore adjusted for education, smoking, age at first birth and BMI, but residual confounding from other potential risk factors is a possibility, and it remains challenging to fully tease apart the effects of chronological age, reproductive age, and age at menopause.

## Conclusions

Our findings suggest that reproductive ageing in women is associated with the mid-life decline in physical function, over and above chronological age in women, but this does not appear to be due to changes in the three reproductive hormones measured as women go through the menopausal transition. Further research on the impact that the menopausal transition might have on physical activity are required to understand whether interventions to maintain physical activity at this stage are valuable for maintaining physical function.

## Supplementary Information

**Additional file 1. Supplementary text.** Physical function composite score. **Figure S1.** Sensitivity analysis of physical function by time since FMP, chronological age, and reproductive hormones with additional adjustment for BMI at baseline. **Figure S2.** Sensitivity analysis of physical function by time since FMP, chronological age, and reproductive hormones with additional adjustment for time-varying BMI. **Figure S3.** Sensitivity analysis of physical function by time since FMP, chronological age, and hormones with additional adjustment for previous test exposure. **Figure S4. **Sensitivity analysis of physical function by time since FMP, chronological age, and reproductive hormones in women who participated in all three follow-up clinic assessments. **Figure S5.** Sensitivity analysis of physical function by time since FMP and chronological age including re-invitations data. **Figure S6. **Sensitivity analysis of physical function by time since FMP and chronological age including re-invitations data additionally adjusted for previous test exposure. **Figure S7.** Sensitivity analysis of physical function by pre-, peri- and post-menopausal stage across chronological age. **Table S1.** Physical function by time since FMP and chronological age. **Table S2.** Model fit according to BIC and QIC. **Table S3.** Physical function by reproductive hormones. **Table S4.** Physical function mean scores at the 1st clinic assessment by follow-up participation. **Table S5. **Physical function mean scores at the 2nd clinic assessment by follow-up participation. **Table S6.** Sensitivity analyses of physical function by reproductive and chronological age in women who participated in all three follow-up clinic assessments. **Table S7.** Sensitivity analyses of physical function by reproductive hormones in women who participated in all three follow-up clinic assessments. **Table S8.** Sensitivity analyses of physical function by reproductive and chronological age including re-invitations data. **Table S9.** Sensitivity analysis of physical function by menopausal stage and chronological age.

## Data Availability

The datasets analysed during the current study and all ALSPAC data are available for analyses by any scientist upon application. Full details can be found at: http://www.bristol.ac.uk/alspac/researchers/access/. For analysis script, please contact the corresponding authors at fanny.kilpi@bristol.ac.uk.

## Abbreviations

AMH: Anti-Müllerian hormone
BMI: Body mass index
FMP: Final menstrual period
FSH: Follicle-stimulating hormone
GEE: Generalised estimating equations
LH: Luteinizing hormone
MLM: Multilevel model
RS: Random slope
