# Supplementary material for: Changes in women’s physical function in mid-life by reproductive age and hormones: a longitudinal study
Source: BMC Womens Health. 2022 Nov 24;22:473. doi: 10.1186/s12905-022-02070-9 (PMC9700972; doi:10.1186/s12905-022-02070-9)
Supplement: Supplementary file 1 — Additional file 1. Supplementary text. Physical function composite score. Figure S1. Sensitivity analysis of physical function by time since FMP, chronological age, and reproductive hormones with additional adjustment for BMI at baseline. Figure S2. Sensitivity analysis of physical function by time since FMP, chronological age, and reproductive hormones with additional adjustment for time-varying BMI. Figure S3. Sensitivity analysis of physical function by time since FMP, chronological age, and hormones with additional adjustment for previous test exposure. Figure S4. Sensitivity analysis of physical function by time since FMP, chronological age, and reproductive hormones in women who participated in all three follow-up clinic assessments. Figure S5. Sensitivity analysis of physical function by time since FMP and chronological age including re-invitations data. Figure S6. Sensitivity analysis of physical function by time since FMP and chronological age including re-invitations data additionally adjusted for previous test exposure. Figure S7. Sensitivity analysis of physical function by pre-, peri- and post-menopausal stage across chronological age. Table S1. Physical function by time since FMP and chronological age. Table S2. Model fit according to BIC and QIC. Table S3. Physical function by reproductive hormones. Table S4. Physical function mean scores at the 1st clinic assessment by follow-up participation. Table S5. Physical function mean scores at the 2nd clinic assessment by follow-up participation. Table S6. Sensitivity analyses of physical function by reproductive and chronological age in women who participated in all three follow-up clinic assessments. Table S7. Sensitivity analyses of physical function by reproductive hormones in women who participated in all three follow-up clinic assessments. Table S8. Sensitivity analyses of physical function by reproductive and chronological age including re-invitations data. Table S9. Sensitivity analysis of physical f [file 12905_2022_2070_MOESM1_ESM.docx]

**ADDITIONAL FILE 1**

**Supplementary Text**

*Physical function composite score*

The composite score of physical function is a combined measure based on three administered tests: grip strength, eyes closed one-leg stand and chair rises. Scores on each of these tests were rescaled to lie between zero (worst) and one (best). Grip strength was divided by participant height, and then divided by the 99^th^ percentile at the first clinic. The value was replaced by 1 if adjusted grip strength was higher than the 99^th^ percentile and coded as 0 if the participant was unable to perform the test. To rescale chair rise time, the 99^th^ percentile of chair rise time at the first clinic was derived. The equation to rescale the score was 1-(chair rise time/99^th^ percentile), which was replaced by 0 if the participant was unable to perform the test or took longer or same time as the 99^th^ percentile. To rescale the eyes closed one-leg stand time, it was divided with the maximum possible time (30s) and replaced by 0 if unable to perform test. The composite score was derived by adding together the three rescaled scores. We standardized the composite score using the first clinic mean and the estimated between-individual SD from the adjusted model described below. At the first clinic, this measure was approximately normally distributed and ranged from 0.12 to 2.60 with a mean of 1.33 (SD 0.37).

**Figure S1.** Sensitivity analysis of physical function by time since FMP, chronological age, and reproductive hormones with additional adjustment for BMI at baseline


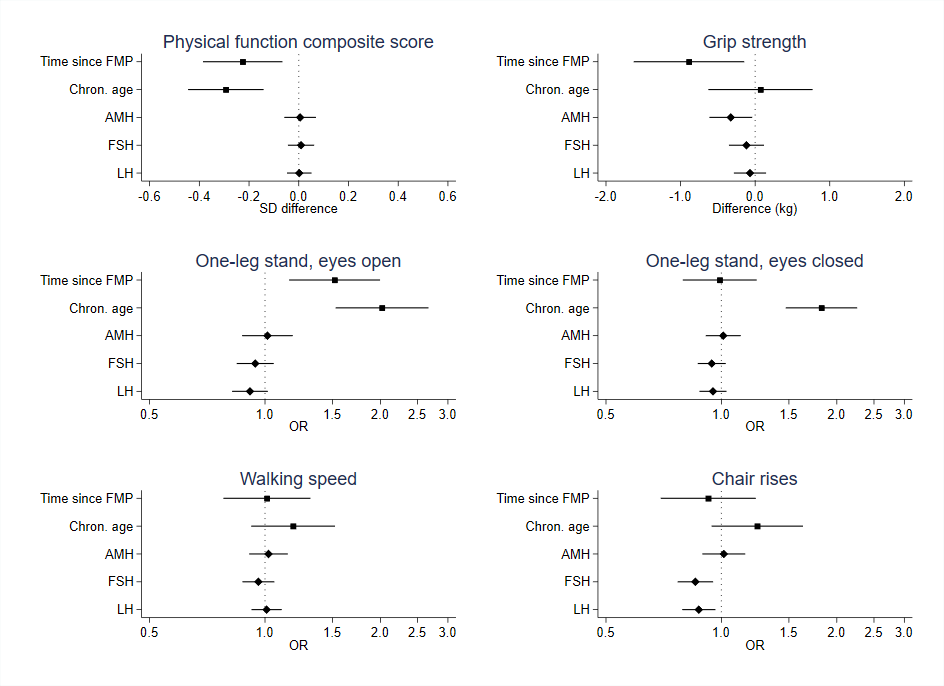


Note: Effect estimates for time since (by 10 years), chronological age centered at 50 (by 10 years), and reproductive hormones from models adjusted for age, education, age at first birth, smoking and BMI at baseline. Positive effect estimates for physical function and grip strength reflect better functioning, whereas positive OR for one-leg stand, walking speed and chair rises indicate worse functioning. AMH: anti-Müllerian hormone; BMI: body mass index; FMP: final menstrual period; FSH: follicle-stimulating hormone; LH: luteinizing hormone; OR: odds ratio; SD: standard deviation.

**Figure S2.** Sensitivity analysis of physical function by time since FMP, chronological age, and reproductive hormones with additional adjustment for time-varying BMI


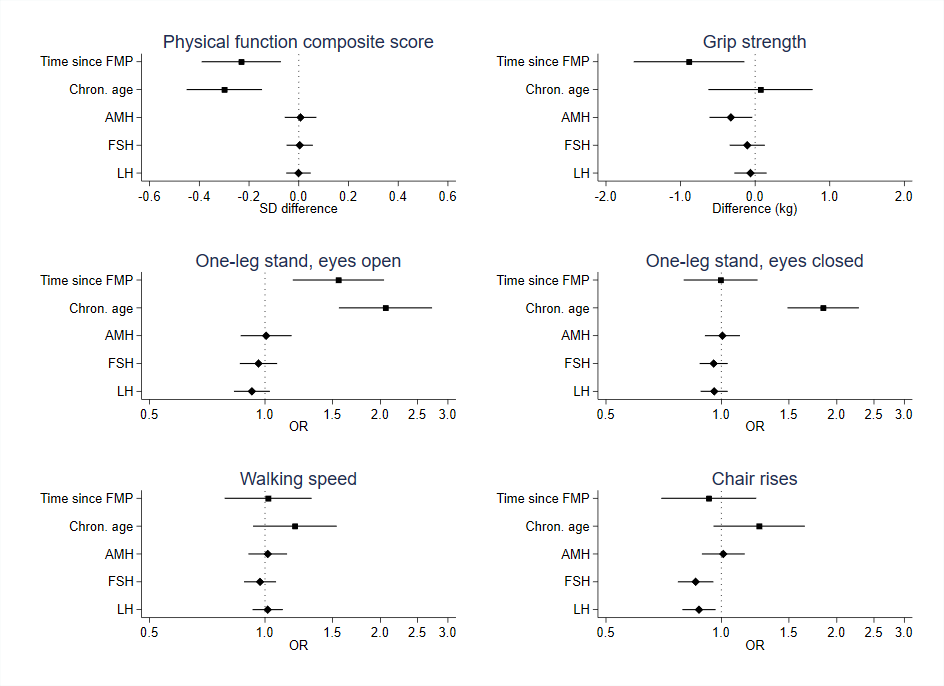


Note: Effect estimates for time since FMP (by 10 years), chronological age centered at 50 (by 10 years), and reproductive hormones from models adjusted for age, education, age at first birth, smoking and time-varying BMI. Positive effect estimates for physical function and grip strength reflect better functioning, whereas positive OR for one-leg stand, walking speed and chair rises indicate worse functioning. AMH: anti-Müllerian hormone; BMI: body mass index; FMP: final menstrual period; FSH: follicle-stimulating hormone; LH: luteinizing hormone; OR: odds ratio; SD: standard deviation.

**Figure S3.** Sensitivity analysis of physical function by time since FMP, chronological age, and hormones with additional adjustment for previous test exposure

**
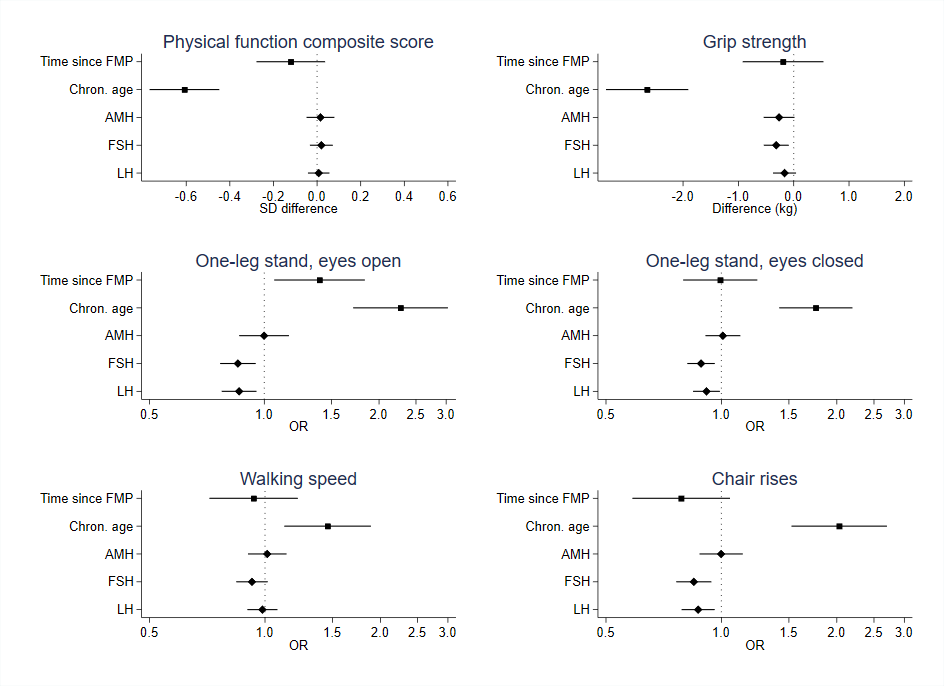
**

Note: Effect estimates for time since FMP (by 10 years), chronological age centered at 50 (by 10 years), and reproductive hormones from models adjusted for age, education, age at first birth, smoking and previous test exposure. Positive effect estimates for physical function and grip strength reflect better functioning, whereas positive OR for one-leg stand, walking speed and chair rises indicate worse functioning. AMH: anti-Müllerian hormone; FMP: final menstrual period; FSH: follicle-stimulating hormone; LH: luteinizing hormone; OR: odds ratio; SD: standard deviation.

**Figure S4.** Sensitivity analysis of physical function by time since FMP, chronological age, and reproductive hormones in women who participated in all three follow-up clinic assessments

**
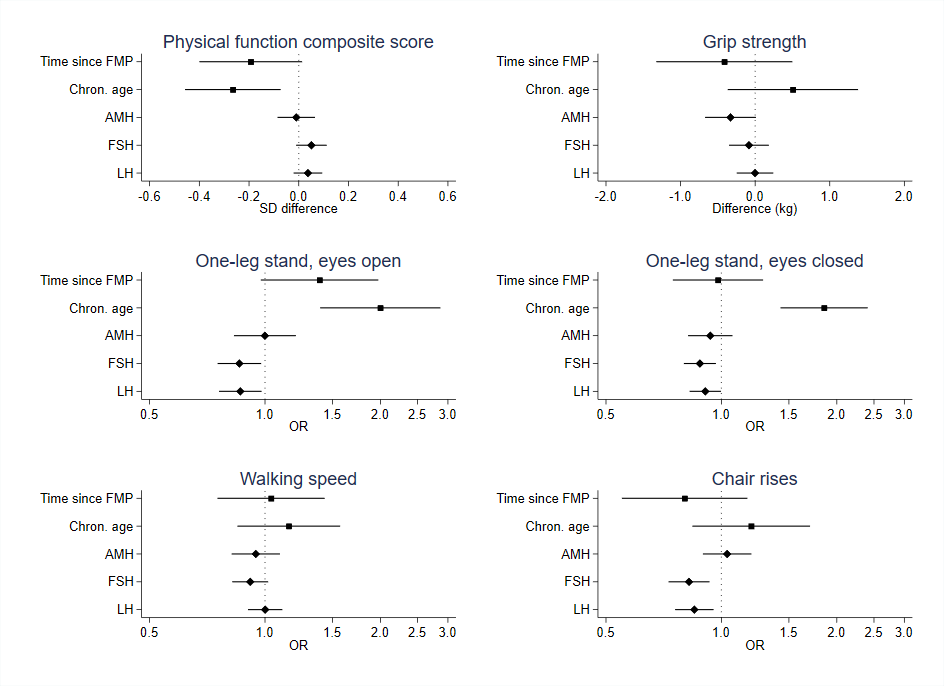
**

Note: Effect estimates for time since FMP (by 10 years), chronological age centered at 50 (by 10 years), and reproductive hormones from models adjusted for age, education, age at first birth and smoking. Positive effect estimates for physical function and grip strength reflect better functioning, whereas positive OR for one-leg stand, walking speed and chair rises indicate worse functioning. AMH: anti-Müllerian hormone; FMP: final menstrual period; FSH: follicle-stimulating hormone; LH: luteinizing hormone; OR: odds ratio; SD: standard deviation.

**Figure S5.** Sensitivity analysis of physical function by time since FMP and chronological age including re-invitations data

**
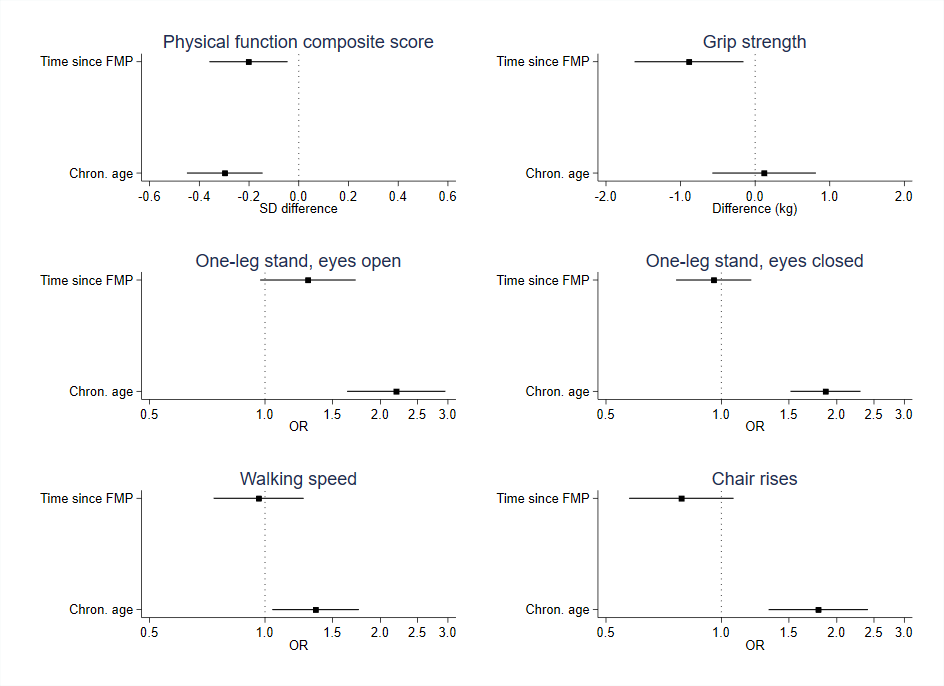
**

Note: Effect estimates for time since FMP (by 10 years), and chronological age centered at 50 (by 10 years) from models adjusted for age, education, age at first birth and smoking. Positive effect estimates for physical function and grip strength reflect better functioning, whereas positive OR for one-leg stand, walking speed and chair rises indicate worse functioning. FMP: final menstrual period; OR: odds ratio; SD: standard deviation.

**Figure S6.** Sensitivity analysis of physical function by time since FMP and chronological age including re-invitations data additionally adjusted for previous test exposure

**
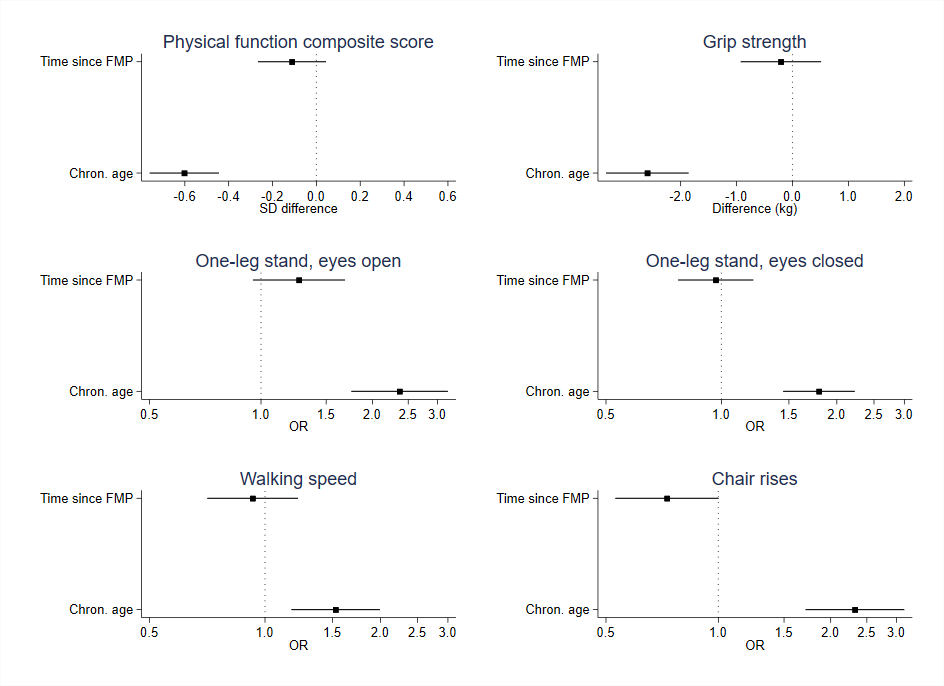
**

Note: Effect estimates for time since FMP (by 10 years), and chronological age centered at 50 (by 10 years) from models adjusted for education, age at first birth, smoking and previous test exposure. Positive effect estimates for physical function and grip strength reflect better functioning, whereas positive OR for one-leg stand, walking speed and chair rises indicate worse functioning. FMP: final menstrual period; OR: odds ratio; SD: standard deviation.

**Figure S7.** Sensitivity analysis of physical function by pre-, peri- and post-menopausal stage across chronological age


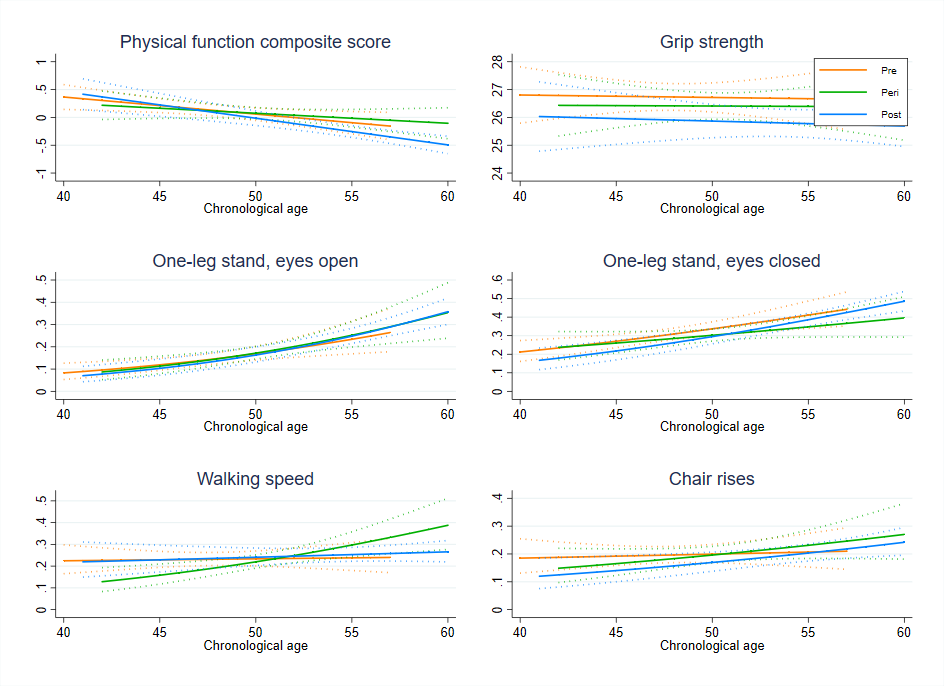


Note: Physical function by menopausal stage across chronological age from a model adjusted for menopausal stage, chronological age, an interaction between chronological age and menopausal stage, education, age at first birth, and smoking. Y axis for physical function reflects z-score for physical function, kg for grip strength and predicted probabilities of low function for one-leg stands, walking speed and chair rises, i.e. greater values for physical function and grip strength reflect better functioning, whereas greater values for one-leg stand, walking speed and chair rises indicate worse functioning.

**Table S1.** Physical function by time since FMP and chronological age

|  | **Time since FMP (per 10 years)** | **Chronological age (per 10 years)** |
| --- | --- | --- |
| *MLM models* |  |  |
| **Physical function composite score** | **SD difference** | **SD difference** |
| Model 1 | -0.32 (-0.46, -0.18) |  |
| Model 2 |  | -0.35 (-0.45, -0.25) |
| Model 3 | -0.29 (-0.45, -0.14) | -0.17 (-0.31, -0.03) |
| Model 4 (adjusted) | -0.21 (-0.37, -0.06) | -0.31 (-0.46, -0.15) |
| Model 4 + baseline BMI | -0.23 (-0.39, -0.07) | -0.29 (-0.45, -0.14) |
| Model 4 + time-varying BMI | -0.23 (-0.39, -0.07) | -0.30 (-0.45, -0.15) |
| Model 4 + previous test exposure | -0.12 (-0.28, 0.04) | -0.61 (-0.77, -0.45) |
| **Grip strength** | **Difference (kg)** | **Difference (kg)** |
| Model 1 | -0.22 (-0.92, 0.47) |  |
| Model 2 |  | -0.47 (-0.94, 0.01) |
| Model 3 | -1.03 (-1.78, -0.29) | 0.11 (-0.53, 0.75) |
| Model 4 (adjusted) | -0.89 (-1.63, -0.15) | 0.08 (-0.62, 0.78) |
| Model 4 + baseline BMI | -0.89 (-1.63, -0.15) | 0.07 (-0.63, 0.77) |
| Model 4 + time-varying BMI | -0.89 (-1.63, -0.14) | 0.07 (-0.63, 0.77) |
| Model 4 + previous test exposure | -0.19 (-0.92, 0.54) | -2.65 (-3.39, -1.91) |
| GEE models |  |  |
| **One-leg stand, eyes open <30s** | **OR** | **OR** |
| Model 1 | 2.14 ( 1.66, 2.75) |  |
| Model 2 |  | 2.01 (1.66, 2.42) |
| Model 3 | 1.61 (1.23, 2.11) | 1.47 (1.14, 1.90) |
| Model 4 (adjusted) | 1.44 (1.10, 1.89) | 2.07 (1.57, 2.74) |
| Model 4 + baseline BMI | 1.52 (1.16, 2.00) | 2.02 (1.53, 2.67) |
| Model 4 + time-varying BMI | 1.55 (1.18, 2.04) | 2.06 (1.56, 2.73) |
| Model 4 + previous test exposure | 1.40 (1.06, 1.84) | 2.28 (1.71, 3.03) |
| **One-leg stand, eyes closed <3s** |  |  |
| Model 1 | 1.42 (1.15, 1.76) |  |
| Model 2 |  | 1.42 (1.23, 1.63) |
| Model 3 | 1.06 (0.85, 1.31) | 1.37 (1.13, 1.66) |
| Model 4 (adjusted) | 0.98 (0.78, 1.22) | 1.86 (1.50, 2.30) |
| Model 4 + baseline BMI | 0.99 (0.79, 1.24) | 1.82 (1.47, 2.26) |
| Model 4 + time-varying BMI | 1.00 (0.80, 1.24) | 1.84 (1.49, 2.28) |
| Model 4 + previous test exposure | 0.99 (0.79, 1.24) | 1.76 (1.42, 2.20) |
| **Walking speed <1.1 m/s** |  |  |
| Model 1 | 1.14 (0.88, 1.47) |  |
| Model 2 |  | 1.10 (0.93, 1.30) |
| Model 3 | 1.08 (0.84, 1.41) | 1.05 (0.84, 1.31) |
| Model 4 (adjusted) | 1.00 (0.77, 1.30) | 1.21 (0.94, 1.55) |
| Model 4 + baseline BMI | 1.01 (0.78, 1.31) | 1.18 (0.92, 1.52) |
| Model 4 + time-varying BMI | 1.02 (0.78, 1.32) | 1.20 (0.93, 1.54) |
| Model 4 + previous test exposure | 0.93 (0.72, 1.22) | 1.46 (1.12, 1.89) |
| **Chair rises >26s** |  |  |
| Model 1 | 1.14 (0.87, 1.49) |  |
| Model 2 |  | 1.21 (1.01, 1.45) |
| Model 3 | 1.03 (0.78, 1.36) | 1.19 (0.93, 1.52) |
| Model 4 (adjusted) | 0.92 (0.69, 1.22) | 1.25 (0.95, 1.64) |
| Model 4 + baseline BMI | 0.92 (0.69, 1.23) | 1.24 (0.94, 1.63) |
| Model 4 + time-varying BMI | 0.93 (0.70, 1.23) | 1.25 (0.95, 1.65) |
| Model 4 + previous test exposure | 0.79 (0.59, 1.05) | 2.03 (1.52, 2.70) |

Note: Model 1: time since FMP (RS) & pre-FMP. Model 2: chronological age (RS). Model 3: time since FMP (RS), chronological age (RS). Model 4: time since FMP (RS), chronological age (RS), education, age at first birth and smoking. BMI: body-mass index; FMP: final menstrual period; GEE: generalized estimating equations; MLM: multilevel model; OR: odds ratio; RS: random slope; SD: standard deviation.

**Table S2.** Model fit according to BIC and QIC

|  | **Physical function** | **Grip strength** | **One leg-stand, eyes open** | **One leg-stand, eyes closed** | **Walking speed** | **Chair rises** |
| --- | --- | --- | --- | --- | --- | --- |
| **Models** | BIC | BIC | QIC | QIC | QIC | QIC |
| Model 1: time since FMP | 18162 | 33818 | 6549 | 4667 | 5539 | 5257 |
| Model 2: chronological age | 18186 | 33874 | 6543 | 4668 | 5543 | 5248 |
| Model 3: time since FMP and chronological age | 18180 | 33854 | 6539 | 4653 | 5538 | 5245 |
| Model 4: adjusted | 18155 | 33862 | 6494 | 4595 | 5512 | 5215 |
| Model 4 + baseline BMI | 18089 | 33871 | 6427 | 4406 | 5450 | 5168 |
| Model 4 + time-varying BMI | 18072 | 33827 | 6402 | 4357 | 5432 | 5159 |
| Model 4 + previous test exposure | 17896 | 33501 | 6492 | 4587 | 5483 | 5086 |

Note: Model 1: time since FMP (RS) & pre-FMP. Model 2: chronological age (RS). Model 3: time since FMP (RS), chronological age (RS). Model 4: time since FMP (RS), chronological age (RS), education, age at first birth and smoking. BIC: Bayesian information criterion; BMI: body-mass index; FMP: final menstrual period; RS: random slope; QIC: quasi-likelihood under the independence model criterion.

**Table S3.** Physical function by reproductive hormones

|  | **AMH** | **FSH** | **LH** |
| --- | --- | --- | --- |
| *MLM models* |  |  |  |
| **Physical function composite score** | **SD difference** | **SD difference** | **SD difference** |
| Model 1 | 0.09 (0.03, 0.15) | -0.05 (-0.10, -0.01) | -0.05 (-0.09, -0.00) |
| Model 2 (adjusted) | 0.00 (-0.06, 0.07) | 0.05 (-0.00, 0.10) | 0.03 (-0.02, 0.08) |
| Model 2 + baseline BMI | 0.00 (-0.06, 0.07) | 0.01 (-0.04, 0.07) | 0.01 (-0.04, 0.05) |
| Model 2 + time-varying BMI | 0.00 (-0.06, 0.07) | 0.01 (-0.05, 0.06) | 0.00 (-0.05, 0.05) |
| Model 2 + previous test exposure | 0.01 (-0.05, 0.08) | 0.02 (-0.03, 0.07) | 0.01 (-0.04, 0.06) |
| **Grip strength (kg)** | **Difference (kg)** | **Difference (kg)** | **Difference (kg)** |
| Model 1 | -0.11 (-0.38, 0.15) | -0.25 (-0.45, -0.05) | -0.19 (-0.39, 0.01) |
| Model 2 (adjusted) | -0.35 (-0.64, -0.05) | -0.13 (-0.36, 0.10) | -0.07 (-0.29, 0.14) |
| Model 2 + baseline BMI | -0.35 (-0.64, -0.05) | -0.12 (-0.35, 0.12) | -0.07 (-0.28, 0.15) |
| Model 2 + time-varying BMI | -0.35 (-0.64, -0.05) | -0.11 (-0.34, 0.13) | -0.06 (-0.27, 0.16) |
| Model 2 + previous test exposure | -0.27 (-0.55, 0.02) | -0.33 (-0.55, -0.10) | -0.17 (-0.38, 0.04) |
| *GEE models* |  |  |  |
| **One-leg stand, eyes open <30s** | **OR** | **OR** | **OR** |
| Model 1 | 0.82 (0.70, 0.96) | 1.04 (0.96, 1.13) | 1.01 (0.93, 1.10) |
| Model 2 (adjusted) | 1.00 (0.86, 1.16) | 0.85 (0.76, 0.94) | 0.86 (0.77, 0.95) |
| Model 2 + baseline BMI | 1.01 (0.87, 1.18) | 0.94 (0.84, 1.05) | 0.91 (0.82, 1.02) |
| Model 2 + time-varying BMI | 1.01 (0.86, 1.17) | 0.96 (0.86, 1.08) | 0.92 (0.83, 1.03) |
| Model 2 + previous test exposure | 1.00 (0.86, 1.16) | 0.85 (0.77, 0.95) | 0.86 (0.77, 0.95) |
| **One-leg stand, eyes closed <3s** |  |  |  |
| Model 1 | 0.92 (0.83, 1.01) | 1.00 (0.94, 1.07) | 1.00 (0.94, 1.08) |
| Model 2 (adjusted) | 1.01 (0.91, 1.12) | 0.89 (0.82, 0.97) | 0.92 (0.85, 1.00) |
| Model 2 + baseline BMI | 1.01 (0.91, 1.12) | 0.94 (0.87, 1.03) | 0.95 (0.88, 1.03) |
| Model 2 + time-varying BMI | 1.01 (0.91, 1.12) | 0.95 (0.88, 1.04) | 0.96 (0.88, 1.04) |
| Model 2 + previous test exposure | 1.01 (0.91, 1.12) | 0.88 (0.81, 0.96) | 0.91 (0.84, 0.99) |
| **Walking speed >1.1m/s** |  |  |  |
| Model 1 | 0.97 (0.87, 1.07) | 0.98 (0.91, 1.06) | 1.02 (0.94, 1.10) |
| Model 2 (adjusted) | 1.02 (0.91, 1.14) | 0.91 (0.83, 1.00) | 0.97 (0.89, 1.07) |
| Model 2 + baseline BMI | 1.02 (0.91, 1.15) | 0.96 (0.87, 1.06) | 1.01 (0.92, 1.11) |
| Model 2 + time-varying BMI | 1.02 (0.91, 1.14) | 0.97 (0.88, 1.07) | 1.02 (0.93, 1.11) |
| Model 2 + previous test exposure | 1.01 (0.90, 1.14) | 0.92 (0.84, 1.02) | 0.98 (0.90, 1.08) |
| **Chair rises >26s** |  |  |  |
| Model 1 | 0.95 (0.84, 1.07) | 0.92 (0.85, 1.00) | 0.92 (0.85, 1.00) |
| Model 2 (adjusted) | 1.01 (0.89, 1.15) | 0.82 (0.74, 0.91) | 0.85 (0.77, 0.94) |
| Model 2 + baseline BMI | 1.01 (0.89, 1.15) | 0.86 (0.77, 0.95) | 0.87 (0.79, 0.96) |
| Model 2 + time-varying BMI | 1.01 (0.89, 1.15) | 0.86 (0.77, 0.95) | 0.87 (0.79, 0.97) |
| Model 2 + previous test exposure | 1.00 (0.88, 1.14) | 0.85 (0.76, 0.94) | 0.87 (0.79, 0.96) |

Note: Model 1: hormone (unadjusted). Model 2: hormone, chronological age (RS), education, age at first birth and smoking. AMH: Anti-Müllerian hormone; BMI: body-mass index; FSH: follicle-stimulating hormone; GEE: generalized estimating equations, LH: luteinizing hormone; MLM: multilevel model; OR: odds ratio; RS: random slope; SD: standard deviation.

**Table S4.** Physical function mean scores at the 1^st^ clinic assessment by follow-up participation

|  | **Mean** | **Mean difference** | **95% CI** |
| --- | --- | --- | --- |
| **Physical function composite score (SD)** |  |  |  |
| All clinics | 0.03 | ref |  |
| 1st and 2nd clinic | -0.16 | -0.19 | -0.39,0.01 |
| 1st and 3rd clinic | -0.17 | -0.21 | -0.57,0.16 |
| Only 1st clinic | 0.01 | -0.02 | -0.21,0.17 |
| **Grip strength (kg)** |  |  |  |
| All clinics | 26.14 | ref |  |
| 1st and 2nd clinic | 25.54 | -0.60 | -1.53,0.34 |
| 1st and 3rd clinic | 25.36 | -0.78 | -2.44,0.88 |
| Only 1st clinic | 26.23 | 0.09 | -0.78,0.96 |
| **One-leg stand, eyes open (s)** |  |  |  |
| All clinics | 27.94 | ref |  |
| 1st and 2nd clinic | 27.98 | 0.05 | -0.78,0.88 |
| 1st and 3rd clinic | 27.10 | -0.84 | -2.32,0.65 |
| Only 1st clinic | 27.50 | -0.43 | -1.21,0.34 |
| **One-leg stand, eyes closed (s)** |  |  |  |
| All clinics | 8.33 | ref |  |
| 1st and 2nd clinic | 7.90 | -0.43 | -1.55,0.70 |
| 1st and 3rd clinic | 7.55 | -0.78 | -2.80,1.24 |
| Only 1st clinic | 8.19 | -0.14 | -1.19,0.91 |
| **3m walk (s)** |  |  |  |
| All clinics | 2.52 | ref |  |
| 1st and 2nd clinic | 2.45 | -0.08 | -0.24,0.08 |
| 1st and 3rd clinic | 2.47 | -0.05 | -0.34,0.24 |
| Only 1st clinic | 2.58 | 0.06 | -0.09,0.20 |
| **10 chair rises (s)** |  |  |  |
| All clinics | 23.62 | ref |  |
| 1st and 2nd clinic | 23.77 | 0.15 | -0.87,1.18 |
| 1st and 3rd clinic | 23.27 | -0.35 | -2.18,1.48 |
| Only 1st clinic | 23.58 | -0.03 | -0.98,0.92 |

Note: CI: confidence interval. SD: standard deviation.

**Table S5.** Physical function mean scores at the 2^nd^ clinic assessment by follow-up participation

|  | **Mean** | **Mean difference** | **95% CI** |
| --- | --- | --- | --- |
| **Physical function composite score (SD)** |  |  |  |
| 2nd and 3rd clinic | -0.21 | ref |  |
| Only 2nd clinic | -0.03 | 0.18 | -0.22,0.58 |
| **Grip strength (kg)** |  |  |  |
| 2nd and 3rd clinic | 25.15 | ref |  |
| Only 2nd clinic | 25.77 | 0.62 | -0.99,2.23 |
| **One-leg stand, eyes open (s)** |  |  |  |
| 2nd and 3rd clinic | 27.26 | ref |  |
| Only 2nd clinic | 26.59 | -0.68 | -2.65,1.29 |
| **One-leg stand, eyes closed (s)** |  |  |  |
| 2nd and 3rd clinic | 7.40 | ref |  |
| Only 2nd clinic | 7.91 | 0.51 | -1.65,2.67 |
| **3m walk (s)** |  |  |  |
| 2nd and 3rd clinic | 2.45 | ref |  |
| Only 2nd clinic | 2.38 | -0.07 | -0.19,0.05 |
| **10 chair rises (s)** |  |  |  |
| 2nd and 3rd clinic | 23.31 | ref |  |
| Only 2nd clinic | 22.85 | -0.46 | -1.80,0.89 |

Note: CI: confidence interval. SD: standard deviation.

**TABLE S6.** Sensitivity analyses of physical function by reproductive and chronological age in women who participated in all three follow-up clinic assessments

|  | **Time since FMP (per 10 years)** | **Chronological age (per 10 years)** |
| --- | --- | --- |
| *MLM models* |  |  |
| **Physical function composite score** | **SD difference** | **SD difference** |
| Model 1 | -0.23 (-0.39, -0.06) |  |
| Model 2 |  | -0.30 (-0.42, -0.18) |
| Model 3 | -0.27 (-0.47, -0.07) | -0.14 (-0.31, 0.04) |
| Model 4 (adjusted) | -0.19 (-0.40, 0.01) | -0.27 (-0.46, -0.07) |
| Model 4 + baseline BMI | -0.21 (-0.42, 0.00) | -0.26 (-0.45, -0.07) |
| Model 4 + time-varying BMI | -0.22 (-0.43, -0.01) | -0.26 (-0.45, -0.07) |
| **Grip strength** | **Difference (kg)** | **Difference (kg)** |
| Model 1 | 0.45 (-0.38, 1.28) |  |
| Model 2 |  | -0.06 (-0.66, 0.55) |
| Model 3 | -0.52 (-1.41, 0.37) | 0.22 (-0.58, 1.02) |
| Model 4 (adjusted) | -0.41 (-1.33, 0.50) | 0.51 (-0.37, 1.38) |
| Model 4 + baseline BMI | -0.41 (-1.32, 0.50) | 0.50 (-0.38, 1.37) |
| Model 4 + time-varying BMI | -0.42 (-1.33, 0.50) | 0.50 (-0.38, 1.37) |
| *GEE models* |  |  |
| **One-leg stand, eyes open <30s** | **OR** | **OR** |
| Model 1 | 2.06 (1.49, 2.84) |  |
| Model 2 |  | 1.97 (1.55, 2.52) |
| Model 3 | 1.56 (1.10, 2.22) | 1.48 (1.06, 2.07) |
| Model 4 (adjusted) | 1.39 (0.97, 1.98) | 2.00 (1.39, 2.87) |
| Model 4 + baseline BMI | 1.45 (1.02, 2.06) | 1.98 (1.38, 2.84) |
| Model 4 + time-varying BMI | 1.50 (1.05, 2.14) | 1.97 (1.37, 2.83) |
| **One-leg stand, eyes closed <3s** |  |  |
| Model 1 | 1.40 (1.08, 1.82) |  |
| Model 2 |  | 1.40 (1.18, 1.67) |
| Model 3 | 1.06 (0.81, 1.39) | 1.35 (1.07, 1.71) |
| Model 4 (adjusted) | 0.98 (0.75, 1.28) | 1.85 (1.43, 2.41) |
| Model 4 + baseline BMI | 0.99 (0.76, 1.30) | 1.83 (1.41, 2.37) |
| Model 4 + time-varying BMI | 1.00 (0.76, 1.31) | 1.83 (1.41, 2.38) |
| **Walking speed <1.1 m/s** |  |  |
| Model 1 | 1.21 (0.89, 1.64) |  |
| Model 2 |  | 1.07 (0.87, 1.31) |
| Model 3 | 1.12 (0.81, 1.54) | 1.00 (0.76, 1.32) |
| Model 4 (adjusted) | 1.04 (0.75, 1.43) | 1.15 (0.85, 1.57) |
| Model 4 + baseline BMI | 1.05 (0.76, 1.44) | 1.15 (0.84, 1.56) |
| Model 4 + time-varying BMI | 1.06 (0.77, 1.46) | 1.15 (0.84, 1.56) |
| **Chair rises >26s** |  |  |
| Model 1 | 0.98 (0.69, 1.38) |  |
| Model 2 |  | 1.16 (0.92, 1.47) |
| Model 3 | 0.90 (0.62, 1.31) | 1.24 (0.91, 1.70) |
| Model 4 (adjusted) | 0.80 (0.55, 1.17) | 1.20 (0.84, 1.70) |
| Model 4 + baseline BMI | 0.80 (0.55, 1.17) | 1.21 (0.85, 1.73) |
| Model 4 + time-varying BMI | 0.81 (0.56, 1.19) | 1.21 (0.85, 1.73) |

Note: Model 1: time since FMP (RS) & pre-FMP. Model 2: chronological age (RS). Model 3: time since FMP (RS), chronological age (RS). Model 4: time since FMP (RS), chronological age (RS), education, age at first birth and smoking. BMI: body mass index; FMP: final menstrual period; GEE: generalized estimating equations; MLM: multilevel model; OR: odds ratio; RS: random slope; SD: standard deviation.

**TABLE S7.** Sensitivity analyses of physical function by reproductive hormones in women who participated in all three follow-up clinic assessments

|  | **AMH** | **FSH** | **LH** |
| --- | --- | --- | --- |
| *MLM models* |  |  |  |
| **Physical function composite score** | **SD difference** | **SD difference** | **SD difference** |
| Model 1 | 0.06 (-0.01, 0.14) | -0.04 (-0.09, 0.01) | -0.03 (-0.08, 0.02) |
| Model 2 (adjusted) | -0.01 (-0.09, 0.06) | 0.05 (-0.01, 0.11) | 0.04 (-0.02, 0.10) |
| Model 2 + baseline BMI | -0.01 (-0.09, 0.06) | 0.02 (-0.04, 0.08) | 0.02 (-0.04, 0.08) |
| Model 2 + time-varying BMI | -0.01 (-0.08, 0.06) | 0.01 (-0.05, 0.07) | 0.01 (-0.04, 0.07) |
| **Grip strength (kg)** | **Difference (kg)** | **Difference (kg)** | **Difference (kg)** |
| Model 1 | -0.16 (-0.46, 0.15) | -0.15 (-0.38, 0.08) | -0.08 (-0.31, 0.15) |
| Model 2 (adjusted) | -0.33 (-0.67, 0.01) | -0.08 (-0.35, 0.18) | -0.00 (-0.25, 0.24) |
| Model 2 + baseline BMI | -0.33 (-0.67, 0.01) | -0.06 (-0.33, 0.21) | 0.01 (-0.24, 0.26) |
| Model 2 + time-varying BMI | -0.33 (-0.67, 0.01) | -0.06 (-0.33, 0.21) | 0.01 (-0.23, 0.26) |
| *GEE models* |  |  |  |
| **One-leg stand, eyes open <30s** | **OR** | **OR** | **OR** |
| Model 1 | 0.82 (0.68, 1.00) | 1.05 (0.95, 1.16) | 1.01 (0.91, 1.13) |
| Model 2 (adjusted) | 1.00 (0.83, 1.20) | 0.86 (0.75, 0.98) | 0.86 (0.76, 0.98) |
| Model 2 + baseline BMI | 1.01 (0.83, 1.23) | 0.97 (0.85, 1.11) | 0.93 (0.81, 1.06) |
| Model 2 + time-varying BMI | 1.00 (0.82, 1.22) | 0.99 (0.86, 1.14) | 0.94 (0.82, 1.07) |
| **One-leg stand, eyes closed <3s** |  |  |  |
| Model 1 | 0.86 (0.76, 0.98) | 0.99 (0.92, 1.07) | 0.99 (0.91, 1.08) |
| Model 2 (adjusted) | 0.94 (0.82, 1.07) | 0.88 (0.80, 0.97) | 0.91 (0.83, 1.00) |
| Model 2 + baseline BMI | 0.94 (0.82, 1.07) | 0.93 (0.84, 1.02) | 0.94 (0.85, 1.03) |
| Model 2 + time-varying BMI | 0.93 (0.82, 1.07) | 0.94 (0.85, 1.04) | 0.95 (0.86, 1.04) |
| **Walking speed >1.1m/s** |  |  |  |
| Model 1 | 0.92 (0.80, 1.05) | 0.97 (0.89, 1.06) | 1.03 (0.94, 1.13) |
| Model 2 (adjusted) | 0.95 (0.82, 1.09) | 0.92 (0.82, 1.02) | 1.00 (0.90, 1.11) |
| Model 2 + baseline BMI | 0.95 (0.82, 1.10) | 0.97 (0.87, 1.08) | 1.04 (0.94, 1.15) |
| Model 2 + time-varying BMI | 0.94 (0.81, 1.09) | 0.98 (0.88, 1.10) | 1.05 (0.94, 1.16) |
| **Chair rises >26s** |  |  |  |
| Model 1 | 0.98 (0.85, 1.12) | 0.93 (0.84, 1.02) | 0.92 (0.83, 1.02) |
| Model 2 (adjusted) | 1.04 (0.89, 1.20) | 0.82 (0.73, 0.93) | 0.85 (0.76, 0.95) |
| Model 2 + baseline BMI | 1.04 (0.90, 1.21) | 0.87 (0.77, 0.99) | 0.88 (0.78, 0.99) |
| Model 2 + time-varying BMI | 1.04 (0.90, 1.21) | 0.88 (0.77, 0.99) | 0.88 (0.78, 0.99) |

Note: Model 1: hormone. Model 2: hormone, chronological age (RS), education, age at first birth and smoking. AMH: Anti-Müllerian hormone; BMI: body mass index; FSH: follicle-stimulating hormone; GEE: generalized estimating equations; LH: luteinizing hormone; MLM: multilevel model; OR: odds ratio; RS: random slope; SD: standard deviation.

**TABLE S8.** Sensitivity analyses of physical function by reproductive and chronological age including re-invitations data

|  | **Time since FMP**  **(per 10 years)** | **Chronological age (per 10 years)** |
| --- | --- | --- |
| *MLM models* |  |  |
| **Physical function composite score** | **SD difference** | **SD difference** |
| Model 1 | -0.30 (-0.43, -0.16) |  |
| Model 2 |  | -0.33 (-0.43, -0.23) |
| Model 3 | -0.28 (-0.44, -0.12) | -0.16 (-0.30, -0.02) |
| Model 4 (adjusted) | -0.20 (-0.36, -0.04) | -0.30 (-0.45, -0.15) |
| Model 4 + baseline BMI | -0.21 (-0.37, -0.05) | -0.28 (-0.44, -0.13) |
| Model 4 + time-varying BMI | -0.22 (-0.38, -0.06) | -0.29 (-0.44, -0.14) |
| Model 4 + previous test exposure | -0.11 (-0.27, 0.04) | -0.60 (-0.76, -0.44) |
| **Grip strength** | **Difference (kg)** | **Difference (kg)** |
| Model 1 | -0.22 (-0.90, 0.47) |  |
| Model 2 |  | -0.44 (-0.91, 0.03) |
| Model 3 | -1.03 (-1.76, -0.29) | 0.15 (-0.49, 0.79) |
| Model 4 (adjusted) | -0.89 (-1.62, -0.16) | 0.12 (-0.57, 0.81) |
| Model 4 + baseline BMI | -0.88 (-1.61, -0.15) | 0.11 (-0.58, 0.81) |
| Model 4 + time-varying BMI | -0.88 (-1.61, -0.15) | 0.11 (-0.58, 0.81) |
| Model 4 + previous test exposure | -0.21 (-0.93, 0.51) | -2.60 (-3.34, -1.86) |
| *GEE models* |  |  |
| **One-leg stand, eyes open <30s** | **OR** | **OR** |
| Model 1 | 1.85 (1.42, 2.42) |  |
| Model 2 |  | 1.96 (1.60, 2.40) |
| Model 3 | 1.46 (1.11, 1.93) | 1.53 (1.17, 2.01) |
| Model 4 (adjusted) | 1.29 (0.97, 1.72) | 2.20 (1.64, 2.95) |
| Model 4 + baseline BMI | 1.36 (1.02, 1.81) | 2.11 (1.57, 2.84) |
| Model 4 + time-varying BMI | 1.39 (1.04, 1.86) | 2.16 (1.60, 2.90) |
| Model 4 + previous test exposure | 1.27 (0.95, 1.69) | 2.37 (1.75, 3.20) |
| **One-leg stand, eyes closed <3s** |  |  |
| Model 1 | 1.37 (1.10, 1.70) |  |
| Model 2 |  | 1.41 (1.22, 1.62) |
| Model 3 | 1.04 (0.84, 1.29) | 1.37 (1.13, 1.66) |
| Model 4 (adjusted) | 0.95 (0.76, 1.20) | 1.87 (1.51, 2.31) |
| Model 4 + baseline BMI | 0.97 (0.77, 1.22) | 1.83 (1.48, 2.25) |
| Model 4 + time-varying BMI | 0.97 (0.78, 1.22) | 1.84 (1.50, 2.27) |
| Model 4 + previous test exposure | 0.97 (0.77, 1.21) | 1.80 (1.45, 2.23) |
| **Walking speed <1.1 m/s** |  |  |
| Model 1 | 1.14 (0.88, 1.48) |  |
| Model 2 |  | 1.17 (0.98, 1.39) |
| Model 3 | 1.05 (0.80, 1.37) | 1.14 (0.91, 1.44) |
| Model 4 (adjusted) | 0.96 (0.73, 1.26) | 1.35 (1.04, 1.76) |
| Model 4 + baseline BMI | 0.98 (0.75, 1.28) | 1.31 (1.01, 1.70) |
| Model 4 + time-varying BMI | 0.99 (0.76, 1.29) | 1.33 (1.02, 1.72) |
| Model 4 + previous test exposure | 0.93 (0.71, 1.22) | 1.53 (1.17, 1.99) |
| **Chair rises >26s** |  |  |
| Model 1 | 1.14 (0.85, 1.53) |  |
| Model 2 |  | 1.47 (1.21, 1.79) |
| Model 3 | 0.89 (0.65, 1.21) | 1.59 (1.21, 2.08) |
| Model 4 (adjusted) | 0.79 (0.57, 1.08) | 1.79 (1.33, 2.41) |
| Model 4 + baseline BMI | 0.80 (0.58, 1.09) | 1.74 (1.29, 2.34) |
| Model 4 + time-varying BMI | 0.80 (0.59, 1.09) | 1.76 (1.31, 2.37) |
| Model 4 + previous test exposure | 0.73 (0.53, 1.00) | 2.32 (1.71, 3.15) |

Note: Model 1: time since FMP (RS) & pre-FMP. Model 2: chronological age (RS). Model 3: time since FMP (RS), chronological age (RS). Model 4: time since FMP (RS), chronological age (RS), education, age at first birth and smoking. BMI: body mass index; FMP: final menstrual period; GEE: generalized estimating equations; MLM: multilevel model; OR: odds ratio; RS: random slope; SD: standard deviation.

**TABLE S9.** Sensitivity analysis of physical function by menopausal stage and chronological age

| **Physical function composite score** | **SD difference** |
| --- | --- |
| Pre-menopause | Ref |
| Peri-menopause | 0.02 (-0.09, 0.12) |
| Post-menopause | -0.07 (-0.22, 0.07) |
| Pre-menopausal chronological age (per 10 years) | -0.31 (-0.55, -0.06) |
| Peri-menopausal chronological age (per 10 years) | -0.18 (-0.45, 0.09) |
| Post-menopausal chronological age (per 10 years) | -0.48 (-0.68, -0.28) |
| **Grip strength** | **Difference (kg)** |
| Pre-menopause | Ref |
| Peri-menopause | -0.31 (-0.76, 0.14) |
| Post-menopause | -0.85 (-1.47, -0.23) |
| Pre-menopausal chronological age (per 10 years) | -0.09 (-1.19, 1.02) |
| Peri-menopausal chronological age (per 10 years) | -0.03 (-1.20, 1.14) |
| Post-menopausal chronological age (per 10 years) | -0.18 (-1.07, 0.71) |
| **One-leg stand, eyes open <30s** | **OR** |
| Pre-menopause | Ref |
| Peri-menopause | 1.02 (0.80, 1.29) |
| Post-menopause | 0.96 (0.72, 1.28) |
| Pre-menopausal chronological age (per 10 years) | 2.25 (1.33, 3.82) |
| Peri-menopausal chronological age (per 10 years) | 2.64 (1.49, 4.67) |
| Post-menopausal chronological age (per 10 years) | 2.86 (2.02, 4.06) |
| **One-leg stand, eyes closed <3s** |  |
| Pre-menopause | Ref |
| Peri-menopause | 0.85 (0.71, 1.02) |
| Post-menopause | 0.82 (0.66, 1.03) |
| Pre-menopausal chronological age (per 10 years) | 1.89 (1.29, 2.76) |
| Peri-menopausal chronological age (per 10 years) | 1.51 (0.95, 2.40) |
| Post-menopausal chronological age (per 10 years) | 2.26 (1.69, 3.01) |
| **Walking speed <1.1 m/s** |  |
| Pre-menopause | Ref |
| Peri-menopause | 0.92 (0.75, 1.14) |
| Post-menopause | 1.04 (0.81, 1.34) |
| Pre-menopausal chronological age (per 10 years) | 1.05 (0.68, 1.63) |
| Peri-menopausal chronological age (per 10 years) | 2.25 (1.34, 3.76) |
| Post-menopausal chronological age (per 10 years) | 1.14 (0.82, 1.59) |
| **Chair rises >26s** |  |
| Pre-menopause | Ref |
| Peri-menopause | 0.98 (0.80, 1.20) |
| Post-menopause | 0.82 (0.63, 1.07) |
| Pre-menopausal chronological age (per 10 years) | 1.10 (0.70, 1.73) |
| Peri-menopausal chronological age (per 10 years) | 1.52 (0.91, 2.54) |
| Post-menopausal chronological age (per 10 years) | 1.56 (1.10, 2.23) |

Note: Model adjusted for menopausal stage, chronological age, an interaction between chronological age and menopausal stage, education, age at first birth, and smoking.
